# Identification of Boolean Network Models From Time Series Data Incorporating Prior Knowledge

**DOI:** 10.3389/fphys.2018.00695

**Published:** 2018-06-08

**Authors:** Thomas Leifeld, Zhihua Zhang, Ping Zhang

**Affiliations:** Institute of Automatic Control, Technische Universität Kaiserslautern, Kaiserslautern, Germany

**Keywords:** Boolean networks, identification, prior knowledge, time series data, network inference

## Abstract

**Motivation:** Mathematical models take an important place in science and engineering. A model can help scientists to explain dynamic behavior of a system and to understand the functionality of system components. Since length of a time series and number of replicates is limited by the cost of experiments, Boolean networks as a structurally simple and parameter-free logical model for gene regulatory networks have attracted interests of many scientists. In order to fit into the biological contexts and to lower the data requirements, biological prior knowledge is taken into consideration during the inference procedure. In the literature, the existing identification approaches can only deal with a subset of possible types of prior knowledge.

**Results:** We propose a new approach to identify Boolean networks from time series data incorporating prior knowledge, such as partial network structure, canalizing property, positive and negative unateness. Using vector form of Boolean variables and applying a generalized matrix multiplication called the semi-tensor product (STP), each Boolean function can be equivalently converted into a matrix expression. Based on this, the identification problem is reformulated as an integer linear programming problem to reveal the system matrix of Boolean model in a computationally efficient way, whose dynamics are consistent with the important dynamics captured in the data. By using prior knowledge the number of candidate functions can be reduced during the inference. Hence, identification incorporating prior knowledge is especially suitable for the case of small size time series data and data without sufficient stimuli. The proposed approach is illustrated with the help of a biological model of the network of oxidative stress response.

**Conclusions:** The combination of efficient reformulation of the identification problem with the possibility to incorporate various types of prior knowledge enables the application of computational model inference to systems with limited amount of time series data. The general applicability of this methodological approach makes it suitable for a variety of biological systems and of general interest for biological and medical research.

## 1. Introduction

Boolean networks (BNs) are discrete-time systems, whose variables can take only two possible values (i.e., 0 and 1). Since Stuart Kaufman firstly introduced BNs in Kauffman (1969) for qualitative description of gene regulatory interactions, BNs have attracted great attention from many scientists and several results have been proposed, for instance, analysis (Albert and Barabási, 2000) and control (Fauré et al., 2006). An overview can be found in Wang et al. (2012) and a database for established models and compatible tools has been introduced (Naldi et al., 2015).

Mathematical models are important to explain dynamic behavior of a system and to understand the functionality of system components (Grieb et al., 2015) and can help scientists to design model-based targeted therapy and diagnosis (Fumia and Martins, 2013). Hence, the inference of models capturing the relevant behavior of the system is an important topic. The inference can be based on the connection of known biochemical reactions, like BN model for the yeast cell cycle in Davidich and Bornholdt (2008), or on experimental data, if the latter is the case it is also called the identification problem. One of the first approaches to identify a BN was REVEAL which is based on mutual information (Liang et al., 1998). In Akutsu et al. (1999) a similar but less complex approach is presented. Both cannot handle errors in the dataset which was solved in Lähdesmäki et al. (2003). The modeled quantities are not Boolean in the experimental data and need to be binarized first. For the binarization several approaches can be found in the literature ranging from mixture model based clustering (Zhou et al., 2003) to more complex methods where the significance of a jump in the time series is estimated in Hopfensitz et al. (2012). A comparison of some identification and binarization approaches and their combinations can be found in Berestovsky and Nakhleh (2013). Most identification approaches are based on previously binarized data, but there also exist approaches directly based on continuous data (e.g., Karlebach and Shamir, 2012). In Higa et al. (2011) the data is considered as given constraint and the set of systems fulfilling the constraints is searched. This approach was then further improved by reducing the sensitivity to noise in Ouyang et al. (2014). An example of recent research is the identification of Boolean models for transient dynamics after perturbations from time course data with answer set programming (Ostrowski et al., 2016). A BN can simply be extended to a Boolean control network (BCN) by considering manipulated external stimuli as control signal of the network. Recently, a powerful tool called semi-tensor product (STP) of matrices has been proposed in Cheng (2001), which can convert the dynamics of BCNs into a model where all information of the dynamics and the structure of the BCN is contained in two matrices (Cheng et al., 2011a). Using the STP based matrix description of BCN several approaches for identifying BCN have been proposed (Cheng and Zhao, 2011; Fornasini and Valcher, 2014; Zhang et al., 2017a).

However, in general, in order to identify the dynamical model of a BCN from its input and output data, a huge number of data is required (Cheng and Zhao, 2011; Cheng et al., 2011b). Though, in practice, data size is limited by the cost of experiments (Geier et al., 2007). In order to reduce the search space and improve the accuracy of the model, the benefit of biological prior knowledge should be taken into consideration. The prior knowledge is used in different ways either by introducing additional constraints in the optimization (Breindl et al., 2013), or reducing the number of parameters in the optimization (Cheng and Zhao, 2011). In Dorier et al. (2016) and Terfve et al. (2012) genetic algorithms are used to handle the complexity problem of large networks while satisfying prior knowledge network graphs as constraints. However, these approaches to handle prior knowledge are not compatible and the advantages of different types of prior knowledge can not be combined. In the approach proposed in this paper, all different types of prior knowledge can be utilized simultaneously and it can additionally handle hypotheses for interactions, which could be used for researcher bias free distinction between alternative hypotheses. Furthermore existing approaches can not handle the case that at some time instances some measurement values are missing, which cannot be avoided in practice due to the limitation of measuring techniques like mass spectrometry-based proteomics.

In this paper, we consider the identification problem of BCNs utilizing biological prior knowledge. A part of the results was presented at the 56th IEEE Conference on Decision and Control in Melbourne (Zhang et al., 2017b). However, the BCN model considered in Zhang et al. (2017b) contains a general output equation. By applying prediction error method (PEM), a high-dimensional BCN (i.e., 2^*n*^ × 2^*n*+*m*^) cannot be avoided. Different from that, although the handling of unmeasurable processes is considered in this paper, the proposed approach leads to a low-dimensional matrix for PEM. Besides, more prior biological knowledge is considered in the paper, like potential interactions, known attractors and limit cycles. Moreover, it is discussed how to deal with alternative hypotheses for interactions and missing measurement points. The main contributions of this paper are as follows:

- A suitable way to handle the prior knowledge such as known network graph, hypotheses for interactions, canalizing and unateness properties or attractor is introduced. For this purpose the BCN is described by two matrices with unknown parameters as entries. If possible, some parameters are inferred directly. Otherwise, relationships between the parameters are set up.
- An approach to deal with the identification of BCNs, in particular, from noisy measurements and missing data points is proposed. The identification problem of BCNs is formulated as a nonlinear pseudo-Boolean optimization, which can be equivalently transformed into a linear binary optimization problem and then solved efficiently.

The remainder of the paper is organized as follows. Section 2 introduces some fundamental definitions and notations. In Section 3, the identification problem of BCNs addressed in this paper will be formulated. Section 4 introduces a way to utilize prior knowledge in identification procedure. The formulation of identification problem of BCNs as an integer linear programming problem is derived and an example is given in Section 5 to illustrate the approach. Finally, a short discussion on the advantages and limitations of the proposed approach is given in Section 6.

## 2. Preliminaries

In this part, we list some necessary notations, which will be used in the subsequent sections.

1. ¬, ∧ and ∨ denote the logical negation (not), conjunction (and) and disjunction (or), respectively.
2. D:={1,0} and $D^{n}=\underset{n}{\underset{︸}{D\times D\times \cdots \times D}}$.
3. $\Delta_{n}:=\left\{\delta_{n}^{k}|1\leq k\leq n\right\}$, where $\delta_{n}^{k}$ denotes the *k*-th column of the identity matrix *I*_*n*_.
4. For a vector *v* ∈ ℝ^*m*^, its *j*-th entry is denoted by [*v*]_*j*_, *j* = 1, 2, ⋯, *m*.
5. An *n* × *t* matrix *L* is called a logical matrix, if $L=\left[\delta_{n}^{i_{1}}\;\delta_{n}^{i_{2}}\;\cdots \;\delta_{n}^{i_{t}}\right]$, where *i*_1_, *i*_2_, ⋯, *i*_*t*_ ∈ {1, 2, ⋯, *n*}, and we express *L* briefly as *L* = δ_*n*_[*i*_1_*i*_2_ ⋯ *i*_*t*_]. Denote the set of *n* × *t* logical matrices by $L_{n\times t}$. *Col*_*i*_(*M*) denotes the *i*-th column of the matrix *M*.
6. $0_{n}:={\left[\underset{n}{\underset{︸}{0\;0\;\cdots \;0}}\right]}^{\text{T}}$, where the superscript T denotes the transpose.

The concept of the semi-tensor product of matrices (STP) has been introduced by Cheng et al. (2011a). The STP of two matrices *A* ∈ ℝ^*m* × *n*^ and *B* ∈ ℝ^*p* × *q*^ is defined as

(1) $$\begin{array}{r} A⋉B=\left(A⊗I_{l/n}\right)\cdot \left(B⊗I_{l/p}\right) \end{array}$$

where ⊗ is the Kronecker product and *l* = *lcm*{*n, p*} is the least common multiple of *n* and *p*. The following property of the STP will be used in the subsequent sections.

Lemma 1. *Let *X* ∈ ℝ^*m* × 1^ and *Y* ∈ ℝ^*n* × 1^. Then *Y*⋉*X* = *W*_[*m, n*]_ ⋉ *X*⋉*Y*, where *W*_[*m, n*]_ is the swap matrix (Cheng et al., 2011a)*.

So the order of two vectors which are multiplied can be altered by multiplying a suitable matrix from the left, this is also called the pseudo-commutativity of the STP. In the following parts the symbol ⋉ will be omitted.

## 3. Problem formulation

System identification is the determination of a model describing the dynamic behavior of a system based on measured data and known perturbations. In the context of Boolean modeling it is assumed that the transient behavior of the system can be qualitatively described by a finite number of Boolean states and that the interaction of these states can be described by Boolean functions. The perturbations are inputs to the system and cause transient behavior of the interacting states in the system. A measured time series of inputs and states form together the data basis for the identification. Depending on the system which is to be modeled, the states might represent the activity of genes or the abundance of proteins and the perturbations could be a stress like heat or oxygen or a chemical substance. In the following the identification process will be formulated as mathematical optimization problem. Therefore the mathematical model of a BCN needs to be defined first. A Boolean control network (BCN) can be described by the following equations (Cheng and Qi, 2010):

(2) $$\{\begin{array}{ll} X_{1}(t+1) & =f_{1}(X_{1}(t),\cdots ,X_{n}(t),U_{1}(t),\cdots ,U_{m}(t))\\ \; & \vdots \\ X_{n}(t+1) & =f_{n}(X_{1}(t),\cdots ,X_{n}(t),U_{1}(t),\cdots ,U_{m}(t)) \end{array}$$

where $X\left(t\right)={\left[X_{1}\left(t\right)\;X_{2}\left(t\right)\;\cdots \;X_{n}\left(t\right)\right]}^{\text{T}}\in D^{n}$, $U\left(t\right)={\left[U_{1}\left(t\right)\;U_{2}\left(t\right)\;\cdots \;U_{m}\left(t\right)\right]}^{\text{T}}\in D^{m}$ are, respectively, the state vector, input vector at time *t*, *f*_*i*_ are logic functions. At the discrete time instances *t* the state variables are updated synchronously according to the logic functions *f*_*i*_. As shown in Cheng and Qi (2010), a vector form of Boolean variable *X*_*i*_, *i* = 1, 2, ⋯, *n* can be simply expressed as

(3) $$\begin{array}{r} x_{i}=\left[\begin{array}{c} X_{i}\\ \neg X_{i} \end{array}\right]. \end{array}$$

Let $x={⋉}_{i=1}^{n}x_{i}\in \Delta_{2^{n}},u={⋉}_{i=1}^{m}u_{i}\in \Delta_{2^{m}}$. According to Cheng and Qi (2010), (2) can be equivalently represented in a vector form:

(4) $$\{\begin{array}{l} x_{1}(t+1)=S_{1}u(t)x(t)\\ \;\vdots \\ x_{n}(t+1)=S_{n}u(t)x(t) \end{array},$$

where $S_{i}\in L_{2\times 2^{n+m}},i=1,2,\cdots \;,n$ are logical matrices. Multiplying all Equations in (4) together, there is

(5) $$\begin{array}{r} x\left(t+1\right)=Lu\left(t\right)x\left(t\right) \end{array}$$

where $L\in L_{2^{n}\times 2^{n+m}}$ is a logical matrix and $Col_{i}\left(L\right)={⋉}_{j=1}^{n}Col_{i}\left(S_{j}\right),i=1,2,\cdots \;,2^{n+m}$.

A polynomial $P_{ml}:{\mathbb{R}}^{k}\to \mathbb{R}$ with *k* variables {θ_1_, θ_2_, ⋯, θ_*k*_} is called multi-linear polynomial, if its degree in each variable is at most 1 (Alon et al., 1991). So, a multi-linear polynomial can be generally expressed as

(6) $$\begin{array}{r} P_{ml}\left(\theta_{1},\theta_{2},\cdots \;,\theta_{k}\right)=c+\overset{k}{\underset{i=1}{\sum }}c_{i}\theta_{i}+\overset{q}{\underset{\alpha =1}{\sum }}c_{I_{\alpha }}\underset{j\in I_{\alpha }}{\prod }\theta_{j} \end{array}$$

where $c,c_{i},c_{I_{\alpha }}\in \mathbb{R}$ for $I_{\alpha }\subset V=\left\{1,2,\cdots \;,k\right\}$ and the set $I_{\alpha }$ has a cardinality of at least 2, i.e., $|I_{\alpha }|\geq 2,\alpha =1,2,\cdots \;,q$.

Generally, the identification problem of BCNs can be described as reconstruction of Boolean functions *f*_*i*_, *i* = 1, 2, ⋯, *n* that explain the experimental data as well as possible. Because of equivalent representation of a Boolean function by a logical matrix, the identification problem is reformulated as searching for logical matrices $S_{i}\in L_{2\times 2^{n+m}},i=1,2,\cdots \;,n$ based on the input and measurement state data.

Note that any logical matrix in $L_{2^{a}\times 2^{b}}$ can be expressed by multi-linear polynomials in a binary parameter vector **θ** of dimension *a* · 2^*b*^. For example, any logical matrix in $L_{4\times 8}$ can be expressed by a binary parameter vector $\theta ={\left[\theta_{1}\;\theta_{2}\;\cdots \;\theta_{16}\right]}^{\text{T}}$ as

$$\begin{array}{l} {\left[\begin{array}{cccc} \theta_{1}\cdot \theta_{2} & \theta_{1}\cdot \left(1-\theta_{2}\right) & \left(1-\theta_{1}\right)\cdot \theta_{2} & \left(1-\theta_{1}\right)\cdot \left(1-\theta_{2}\right)\\ \theta_{3}\cdot \theta_{4} & \theta_{3}\cdot \left(1-\theta_{4}\right) & \left(1-\theta_{3}\right)\cdot \theta_{4} & \left(1-\theta_{3}\right)\cdot \left(1-\theta_{4}\right)\\ \theta_{5}\cdot \theta_{6} & \theta_{5}\cdot \left(1-\theta_{6}\right) & \left(1-\theta_{5}\right)\cdot \theta_{6} & \left(1-\theta_{5}\right)\cdot \left(1-\theta_{6}\right)\\ \theta_{7}\cdot \theta_{8} & \theta_{7}\cdot \left(1-\theta_{8}\right) & \left(1-\theta_{7}\right)\cdot \theta_{8} & \left(1-\theta_{7}\right)\cdot \left(1-\theta_{8}\right)\\ \theta_{9}\cdot \theta_{10} & \theta_{9}\cdot \left(1-\theta_{10}\right) & \left(1-\theta_{9}\right)\cdot \theta_{10} & \left(1-\theta_{9}\right)\cdot \left(1-\theta_{10}\right)\\ \theta_{11}\cdot \theta_{12} & \theta_{11}\cdot \left(1-\theta_{12}\right) & \left(1-\theta_{11}\right)\cdot \theta_{12} & \left(1-\theta_{11}\right)\cdot \left(1-\theta_{12}\right)\\ \theta_{13}\cdot \theta_{14} & \theta_{13}\cdot \left(1-\theta_{14}\right) & \left(1-\theta_{13}\right)\cdot \theta_{14} & \left(1-\theta_{13}\right)\cdot \left(1-\theta_{14}\right)\\ \theta_{15}\cdot \theta_{16} & \theta_{15}\cdot \left(1-\theta_{16}\right) & \left(1-\theta_{15}\right)\cdot \theta_{16} & \left(1-\theta_{15}\right)\cdot \left(1-\theta_{16}\right) \end{array}\right]}^{\text{T}} \end{array}$$

where the superscript T denotes the transpose. In this way, each realization of the binary parameter vector $\theta \in D^{a2^{b}}$ corresponds to a unique logical matrix. It is straightforward to equivalently convert this logical matrix into readable logical equations. Based on this, the objective of the paper is to find a binary parameter vector **θ**, such that dynamic behavior of the BCN (5) is consistent with the important dynamics captured in the observed input-state data.

## 4. Incorporation of prior knowledge

In this section, we shall show how to utilize known network graph, potential interactions, canalizing and unateness properties and attractors in the identification procedure.

### 4.1. Known or potential interactions

Often some or all interaction partners are known in a biological system which is subject of identification. This knowledge can come from databases or can be constructed based knowledge about the underlying biochemical reactions. In some cases a known signaling network is to be complemented and different hypothesis for potential interactions shall be evaluated. If all interaction partners and the direction of the interactions are known, the underlying directed network graph of the BN is known.

In graph theory, a directed graph can be denoted by G={V,E}, where V is a finite set of nodes and E⊂V×V is a finite set of edges (Bollobas, 2012). If $\left(v_{i},v_{j}\right)\in E$, then there is an edge from *v*_*i*_ → *v*_*j*_. According to Cheng et al. (2011a), a BCN can be represented by a directed graph, where each gene is considered as a node. If there is an edge from *X*_*i*_ → *X*_*j*_, then *X*_*j*_ is affected by *X*_*i*_.

Assume that a directed graph for a BCN G={V,E} is known. Then we have the following result.

Lemma 2. *If the node *X*_*i*_ is affected by *w* nodes, then 2^*w*^ binary parameters are enough to describe the corresponding logical matrix *S*_*i*_*.

*Proof*. As the node *x*_*i*_ is affected by *w* nodes, then the Boolean function can be represented in a vector form as

$$\begin{array}{l} x_{i}\left(t+1\right)=S_{i}x_{i_{1}}\left(t\right)x_{i_{2}}\left(t\right)\cdots x_{i_{w}}\left(t\right) \end{array}$$

where the matrix *S*_*i*_ is a logical matrix of dimension 2 × 2^*w*^. Recall that the logical matrix *S*_*i*_ is a matrix containing only columns belonging to Δ_2_ (Cheng et al., 2011a). Hence, 2^*w*^ binary parameters are enough for the description of the logical matrix *S*_*i*_.

An example is given below to express logical matrices of a BCN with a known network graph with the help of binary parameters.

**Figure 1:**
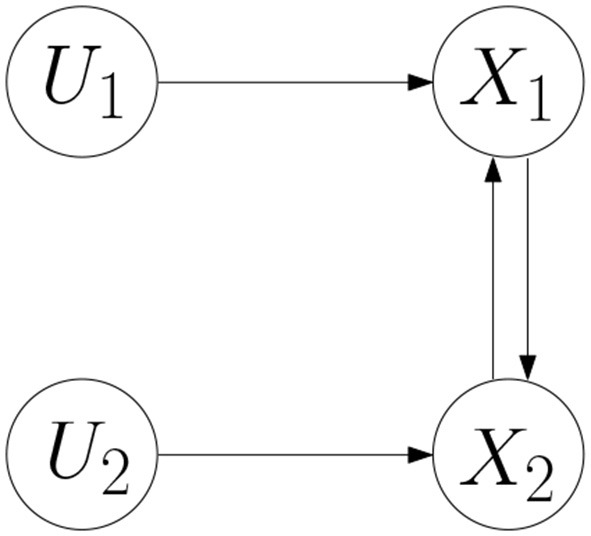
Network graph.

Example 1. Consider a BCN as follows.

(7) $$\{\begin{array}{ll} X_{1}(t+1) & =f_{1}(X_{2}(t),U_{1}(t))\\ X_{2}(t+1) & =f_{2}(X_{1}(t),U_{2}(t)) \end{array}$$

where the network graph of the BCN is shown in Figure 1 (Cheng and Zhao, 2011). According to Cheng and Qi (2010), the algebraic form of the BCN is obtained,

(8) $$\{\begin{array}{l} x_{1}(t+1)=S_{1}u_{1}(t)x_{2}(t)\\ x_{2}(t+1)=S_{2}u_{2}(t)x_{1}(t) \end{array}$$

where the logical matrices $S_{1},S_{2}\in L_{2\times 4}$ can be expressed by the binary parameter vector $\theta ={\left[\theta_{1}\;\theta_{2}\;\cdots \;\theta_{8}\right]}^{\text{T}}$ in the following form:

$$\begin{array}{ll} S_{1} & =\left[\begin{array}{cccc} \theta_{1} & \theta_{2} & \theta_{3} & \theta_{4}\\ 1-\theta_{1} & 1-\theta_{2} & 1-\theta_{3} & 1-\theta_{4} \end{array}\right],\\ S_{2} & =\left[\begin{array}{cccc} \theta_{5} & \theta_{6} & \theta_{7} & \theta_{8}\\ 1-\theta_{5} & 1-\theta_{6} & 1-\theta_{7} & 1-\theta_{8} \end{array}\right]. \end{array}$$

Potential interactions can be treated in the same way as known interactions as long as all of them could potentially be simultaneously true. If there are two alternative hypotheses and the question is which fits better to the data, then this can be done by introducing a constraint on the parameters θ.

Example 2. Assume that *X*_1_ is influenced either by *X*_2_ or by *U*_1_, this could be ensured by imposing the constraint

(9) $$\begin{array}{r} \lambda \left(\theta_{1}-\theta_{2}\right)\cdot \left(\theta_{3}-\theta_{4}\right)+\left(1-\lambda \right)\left(\theta_{1}-\theta_{3}\right)\cdot \left(\theta_{2}-\theta_{4}\right)=0,\;\lambda \in \left\{0,1\right\}, \end{array}$$

### 4.2. Canalizing boolean functions

The concept of “canalizing” values in Boolean functions was introduced in developmental biology in 1940s (Waddington, 1942). The idea is, that one input is dominant and if it takes a certain value it determines the output. After that, in order to explain the phenomenon that absence of repressor or high levels of allolactose assures the operator cannot bind repressor in *lac operon* of the bacterium *Escherichia coli*, Kauffman applied this concept to BN modeling of gene regulatory networks (Kauffman, 1974).

Canalizing functions are defined as follows.

*Definition 1*. A Boolean function $f:D^{n}\overset{f}{\underset{\;}{\to }}D$ is canalizing if there exist a variable *X*_*i*_, *i* ∈ {1, 2, ⋯, *n*} and a Boolean function *g*(*X*_1_, ⋯, *X*_*i* − 1_, *X*_*i* + 1_, ⋯, *X*_*n*_) and a,b∈D, such that

(10) $$f(X_{1},\cdots ,X_{n})=\{\begin{array}{ll} b, & \text{if }X_{i}=a,\\ g\neq b, & \text{if }X_{i}\neq a \end{array}$$

where *a* is called the canalizing value for the variable *X*_*i*_ and *b* is the canalizing output value (Kauffman, 1974).

Based on Definition 1, this prior knowledge can be translated into imposing a specified value in the corresponding logical matrix. Assume that the logical matrix for the canalizing function (10) is denoted as *S* and the canalizing value *a* and canalizing output *b* can, respectively, be expressed in a vector form as $\delta_{2}^{2-a}$ and $\delta_{2}^{2-b}$. Then, we can get the following result.

**Theorem 1**. *Given a canalizing function (10). The corresponding logical matrix $S\in L_{2\times 2^{n}}$ satisfies*

(11) $$SW_{\left[2,2^{i-1}\right]}\delta_{2}^{2-a}=\delta_{2}\underset{2^{n-1}}{\underset{︸}{\left[2-b\;\;2-b\;\cdots \;2-b\right].}}$$

where $W_{\left[2,2^{i-1}\right]}$ is the swap matrix.

*Proof*. According to Lemma 1, it is easy to obtain $Sx_{1}x_{2}\cdots x_{n}=SW_{\left[2,2^{i-1}\right]}x_{i}x_{1}x_{2}\cdots x_{i-1}x_{i+1}\cdots x_{n}$. Applying (11), we have

$$\begin{array}{l} SW_{\left[2,2^{i-1}\right]}\delta_{2}^{2-a}x_{1}x_{2}\cdots x_{i-1}x_{i+1}\cdots x_{n}\\ =\delta_{2}\underset{2^{n-1}}{\underset{︸}{\left[2-b\;\;2-b\;\cdots \;2-b\right]}}x_{1}x_{2}\cdots x_{i-1}x_{i+1}\cdots x_{n}=\delta_{2}^{2-b} \end{array}$$

which corresponds to *f*(*X*_1_, ⋯, *X*_*i*−1_, *a, X*_*i*+1_, ⋯, *X*_*n*_) = *b* for any *X*_1_, ⋯, *X*_*i*−1_, *X*_*i*+1_, ⋯, *X*_*n*_ ∈ {0, 1}.

Let's take an example to illustrate the result of Theorem 1.

Example 3. Consider the BCN (7). Assume that the Boolean function *f*_1_ is a canalizing function in *x*_2_ for a canalizing value $\delta_{2}^{2}$ and the corresponding canalizing output is $\delta_{2}^{1}$. Due to the canalizing property, the logical matrix *S*_1_ can be reduced to

$$\begin{array}{l} S_{1}W_{\left[2,2\right]}\delta_{2}^{2}=\left[\begin{array}{cc} 1 & 1\\ 0 & 0 \end{array}\right]\Rightarrow S_{1}=\left[\begin{array}{cccc} \theta_{1} & 1 & \theta_{3} & 1\\ 1-\theta_{1} & 0 & 1-\theta_{3} & 0 \end{array}\right]. \end{array}$$

It can be checked that $S_{1}u_{1}\delta_{2}^{2}=\delta_{2}^{1}$, no matter whether $u_{1}=\delta_{2}^{1}$ or $u_{1}=\delta_{2}^{2}$. Note that the logical matrix *S*_1_ contains only two binary parameters (i.e., θ_1_ and θ_3_). It shows that using canalizing property can reduce the number of binary parameters.

As an important subclass of canalizing function, *k*-canalizing function is defined as follows.

*Definition 2*. Let σ be a permutation on the set {1, 2, ⋯, *n*}. A Boolean function $f:D^{n}\overset{f}{\underset{\;}{\to }}D$ is *k*-canalizing in the variable order *X*_σ(1)_, *X*_σ(2)_, ⋯, *X*_σ(*k*)_ with canalizing input values *a*_1_, *a*_2_, ⋯, *a*_*k*_ and canalizing output values *b*_1_, *b*_2_, ⋯, *b*_*k*_, if it can be represented in the form (Kauffman et al., 2003).

(12) $$f(X_{1},\cdots ,X_{n})\text{​}=\text{​​}\{\begin{array}{ll} b_{1}, & \text{if }X_{\sigma (1)}=a_{1},\\ b_{2}, & \text{if }X_{\sigma (1)}\neq a_{1},X_{\sigma (2)}=a_{2},\\ \; & \vdots \\ b_{k}, & \text{if }X_{\sigma (1)}\neq a_{1},X_{\sigma (2)}\neq a_{2}\cdots \\ \; & \;X_{\sigma (k)}=a_{k},\\ g\neq b_{k}, & \text{if }X_{\sigma (1)}\neq a_{1},X_{\sigma (2)}\neq a_{2}\cdots \\ \; & \;X_{\sigma (k)}\neq a_{k}. \end{array}$$

Note that if all variables have certain canalizing values, then the function is called *nested canalizing function* (Kauffman et al., 2003).

As a Boolean variable can only take two values, i.e., {0, 1}, (12) can be equivalently expressed as *f*(*X*_1_, ⋯, *X*_*n*_) = *b*_*i*_, if *X*_σ(1)_ = 1−*a*_1_, *X*_σ(2)_ = 1−*a*_2_, ⋯, *X*_σ(*i*)_ = *a*_*k*_, *i* = 1, 2, ⋯, *k*. Using the Boolean variables ${\left[X_{\sigma \left(1\right)}X_{\sigma \left(2\right)}\cdots X_{\sigma \left(i\right)}\right]}^{\text{T}}$ to represent a multi-valued logic variable, it is straightforward to recognize that a *k*-canalizing function can be equivalently formulated as a canalizing function in a multi-valued logic variable. Therefore, Theorem 1 can be applied to specify the logical matrix for *k*-canalizing or nested canalizing function (12).

It is necessary to point out that different from the approaches proposed in Breindl et al. (2013) and Faisal et al. (2010), some binary parameters can be directly inferred, no matter which canalizing value the canalizing variable takes.

Example 4. Consider the BCN (7). Assume that the Boolean function *f*_2_ is nested canalizing function, which can be represented as

$$f_{2}(U_{2},X_{1})=\{\begin{array}{ll} 1, & \text{if }U_{2}=1,\\ 0, & \text{if }U_{2}\neq 1,X_{1}=1. \end{array}$$

Because *f*_2_(1, *X*_1_) = 1 for *X*_1_ ∈ {0, 1}, we have

$$\begin{array}{l} S_{2}\delta_{2}^{1}=\left[\begin{array}{cc} 1 & 1\\ 0 & 0 \end{array}\right]\Rightarrow S_{2}=\left[\begin{array}{cccc} 1 & 1 & \theta_{7} & \theta_{8}\\ 0 & 0 & 1-\theta_{7} & 1-\theta_{8} \end{array}\right]. \end{array}$$

Moreover, due to *f*_2_(0, 1) = 0, there is

$$\begin{array}{l} S_{2}\delta_{2}^{2}\delta_{2}^{1}=\left[\begin{array}{c} 0\\ 1 \end{array}\right]\Rightarrow S_{2}=\left[\begin{array}{cccc} 1 & 1 & 0 & \theta_{8}\\ 0 & 0 & 1 & 1-\theta_{8} \end{array}\right]. \end{array}$$

Remark 1. *Theorem 1 implies that considering canalizing property of a Boolean function, the corresponding logical matrix can be expressed with fewer binary parameters. For instance, if a Boolean function *f*(*X*_1_, *X*_2_, ⋯, *X*_*n*_) is a *k*-canalizing function, then 2^*n*−*k*^ different binary parameters are enough to represent the corresponding logical matrix*.

### 4.3. Unate boolean functions

The behavior of some substances or genes are well studied and it is known that they act as suppressing or activating in all reactions they are involved. If they always act inhibiting they have the so called negative unateness property. For the case that a quantity exclusively induces the expression of another gene or substance it has the positive unateness property (Porreca et al., 2010).

For the mathematical modeling of the unatess properties let us consider another important type of Boolean functions, which is called the unate function (Breindl et al., 2013).

*Definition 3*. (Breindl et al., 2013) A Boolean function $f:D^{n}\overset{f}{\underset{\;}{\to }}D$ is unate in *x*_*i*_, if for any ${\left[X_{1}X_{2}\;\cdots X_{i-1}X_{i+1}\;\cdots \;X_{n}\right]}^{\text{T}}\in D^{n-1}$ it holds for positive unateness that

(13) $$\begin{array}{r} f\left(\cdots ,X_{i-1},0,X_{i+1},\cdots \right)\leq f\left(\cdots ,X_{i-1},1,X_{i+1},\cdots \right) \end{array}$$

or it always holds for negative unateness that

(14) $$\begin{array}{r} f\left(\cdots ,X_{i-1},0,X_{i+1},\cdots \right)\geq f\left(\cdots ,X_{i-1},1,X_{i+1},\cdots \right) \end{array}$$

In the same way as Breindl et al. (2013), unateness can be equivalently represented as linear formulation. Afterwards, this linear formulation can be seen as additional inequality constraints in the optimization problem. As Boolean function can be rewritten as a vector form (4) and according to Lemma 1, there is

(15) $$\begin{array}{r} \begin{array}{c} Sx_{1}x_{2}\cdots x_{i-1}x_{i}x_{i+1}\cdots x_{n}=SW_{\left[2,2^{i-1}\right]}x_{i}x_{1}x_{2}\cdots x_{i-1}x_{i+1}\cdots x_{n} \end{array} \end{array}$$

where *S* is the logical matrix corresponding to the Boolean function *f*. Hence, *f*(⋯, *X*_*i*−1_, 0, *X*_*i*+1_, ⋯) and *f*(⋯, *X*_*i*−1_, 1, *X*_*i*+1_, ⋯) can, respectively, be represented in a vector form as

(16) $$\begin{array}{r} SW_{\left[2,2^{i-1}\right]}\delta_{2}^{2}x_{1}x_{2}\cdots x_{i-1}x_{i+1}\cdots x_{n} \end{array}$$

and

(17) $$\begin{array}{r} SW_{\left[2,2^{i-1}\right]}\delta_{2}^{1}x_{1}x_{2}\cdots x_{i-1}x_{i+1}\cdots x_{n} \end{array}$$

Furthermore, based on the vector form of Boolean variable (3) and according to (13) or (14), for each *x*_1_, *x*_2_, ⋯, *x*_*i*−1_, *x*_*i*+1_, ⋯, *x*_*n*_ ∈ Δ_2_ an inequality can be set up. Putting all inequality constraints together, we can find a matrix *A* for the following expression.

(18) $$\begin{array}{r} A\cdot \theta \leq 0_{n} \end{array}$$

Example 5. Consider the Boolean function *x*_1_ = *f*_1_(*x*_2_), this function *f*_1_ is defined by two unknown parameters θ_1_ and θ_2_. Assume that the Boolean function *f*_1_ is a unate function with respect to *x*_2_, which satisfies (13). As the first step, the matrix $S_{1}\delta_{2}^{1}$ and $S_{1}\delta_{2}^{2}$ are calculated, which yields

$$\begin{array}{l} S_{1}\delta_{2}^{1}=\left[\begin{array}{c} \theta_{1}\\ 1-\theta_{1} \end{array}\right],\;S_{1}\delta_{2}^{2}=\left[\begin{array}{c} \theta_{2}\\ 1-\theta_{2} \end{array}\right]. \end{array}$$

Then, the inequality constraint is

$$\begin{array}{l} \theta_{2}\leq \theta_{1}\Leftrightarrow \left[\begin{array}{cc} -1 & 1 \end{array}\right]\cdot \left[\begin{array}{c} \theta_{1}\\ \theta_{2} \end{array}\right]\leq 0. \end{array}$$

### 4.4. Known attractors or limit cycles

When the BCN is not perturbed for a sufficiently long time it reaches the steady state. The steady state of a BCN can be exactly one state (i.e., attractor) or a fix cycle of some states (i.e., limit cycle). Attractors or limit cycles are assumed to determine the phenotype in the cell differentiation (Huang and Ingber, 2000). The experimental setup to measure the steady state of a system is simpler and measurements are easier to reproduce compared with transient dynamics. As a result, the steady state of the BN is often already known when the perturbation experiments for identification of the transient behavior are carried out. This knowledge can be utilized as follows.

An attractor corresponds to a self loop in the reachability graph. For a given input combination this fixes one specific coulumn in the matrix *L*. For the constant input $u\left(t\right)=\delta_{2^{m}}^{i}$ and the constant state $x\left(t\right)=\delta_{2^{n}}^{j}$ the k-th column is known to be $Col_{k}\left(L\right)=\delta_{2^{n}}^{j}$ with *k* = (*i* − 1)2^*n*^ + *j*. A limit cycle can be analyzed in a similar manner. For the given state sequence of the limit cycle of length *T* and the constant input $u\left(t\right)=\delta_{2^{m}}^{i}$ one can calculate *T* columns of *L*. For each time instant *t* of the cycle the actual state $x\left(t\right)=\delta_{2^{n}}^{j}$ and the next state $x\left(t+1\right)=\delta_{2^{n}}^{w}$ is known. The information of this known transition is used by setting the k-th column to $Col_{k}\left(L\right)=\delta_{2^{n}}^{w}$ with *k* = (*i*−1)2^*n*^ + *j*.

## 5. Identification approach

In this part, the identification problem of BCNs will be studied. At first, it will be shown that the identification problem can be reformulated as a nonlinear pseudo-Boolean optimization problem by applying the idea of the prediction error method. The pseudo-Boolean optimization can be transformed into an equivalent linear binary integer programming problem that can be solved more efficiently. Then, we give a way to deal with missing measurement values. Finally, we discuss how dependencies between measured substances can be handled.

### 5.1. Optimization problem

The prediction error method (PEM) is one of the most widely used identification methods (Isermann and Münchhof, 2011). The basic idea behind this method is to choose parameters to make the difference between a prediction based on the model and the measured values as small as possible. As the PEM minimizes the prediction error in the identified system, errors in the data set due to noise need no special treatment. Obviously the more noise is expected in the data set the more data should be acquired for identification of a reliable model.

Before applying PEM, it is necessary to specify a measure of prediction error. In information theory, the Hamming distance *d*(*X, Y*) between two vectors $X,Y\in D^{n}$ is defined as the number of positions, in which the entries differ (Hamming, 1950).

(19) $$\begin{array}{r} d\left(X,Y\right)=|\left\{j\in \left\{1,2,\cdots \;,n\right\}|\;{\left[X\right]}_{j}\neq {\left[Y\right]}_{j}\right\}| \end{array}$$

As each entry in the vectors *X* and *Y* belongs to the Boolean domain {0, 1}, (19) can be equivalently written as

(20) $$\begin{array}{r} d\left(X,Y\right)=\overset{n}{\underset{i=1}{\sum }}{\left|[X\right]}_{i}-{\left[Y]\right|}_{i} \end{array}$$

Furthermore, let *x*_*i*_, *y*_*i*_ be, respectively, the vector form of [*X*]_*i*_ and [*Y*]_*i*_. Then, it is straightforward to get

(21) $$\begin{array}{r} {\left|[X\right]}_{i}-{\left[Y\right]}_{i}|=(1-x_{i}^{\text{T}}\cdot y_{i}) \end{array}$$

Based on this, the Hamming distance *d*(*X, Y*) can be rewritten as

(22) $$\begin{array}{r} d\left(X,Y\right)=\overset{n}{\underset{i=1}{\sum }}(1-x_{i}^{\text{T}}\cdot y_{i}) \end{array}$$

Assume that the observed input and state data is {(*U*(*t*), *X*(*t*)), *t* = 0, 1, ⋯, *T*}. The vector form of the input data {*U*_1_(*t*), *U*_2_(*t*), ⋯, *U*_*m*_(*t*)} and state data {*X*_1_(*t*), *X*_2_(*t*), ⋯, *X*_*n*_(*t*)} are denoted, respectively, as *u*_1_(*t*), *u*_2_(*t*), ⋯, *u*_*m*_(*t*) and *x*_1_(*t*), *x*_2_(*t*), ⋯, *x*_*n*_(*t*). Since the logical matrix *S*_*i*_ for the state variable *X*_*i*_ can be represented by the parameter vector **θ**, we simply denote them as *S*_*i*_(**θ**). Suppose that the state variable *X*_*i*_ can be influenced by the variables *X*_*j*_1__, *X*_*j*_2__, ⋯, *X*_*j*_*k*__. According to (5), it is easy to get expression of the prediction ${\hat{x}}_{i}\left(\theta ,t\right)$:

(23) $$\begin{array}{r} {\hat{x}}_{i}\left(\theta ,t\right)=S_{i}\left(\theta \right)u\left(t-1\right){⋉}_{i=1}^{k}x_{j_{i}}\left(t-1\right) \end{array}$$

Recalling (21) and (22), the PEM method will estimate the binary parameters by minimizing the prediction error, i.e.,

(24) $$\begin{array}{r} \underset{\theta \in D^{k}}{\min}\overset{T}{\underset{t=0}{\sum }}d\left(X\left(t\right),\hat{X}\left(\theta ,t\right)\right)=\underset{\theta \in D^{k}}{\min}\overset{T}{\underset{t=0}{\sum }}\overset{n}{\underset{i=1}{\sum }}(1-x_{i}^{\text{T}}\left(t\right)\cdot {\hat{x}}_{i}\left(\theta ,t)\right) \end{array}$$

Furthermore, the optimization problem (24) can be equivalently rewritten as

$$\underset{\theta \in D^{k}}{\min}\left(T\cdot n-\sum_{t=0}^{T}\sum_{i=1}^{n}x_{i}^{\text{T}}(t)\cdot {\hat{x}}_{i}(\theta ,t)\right)$$

which is actually equivalent to

(25) $$\begin{array}{r} \underset{\theta \in D^{k}}{\max}\overset{T}{\underset{t=0}{\sum }}\overset{n}{\underset{i=1}{\sum }}x_{i}^{\text{T}}\left(t\right)\cdot {\hat{x}}_{i}\left(\theta ,t\right) \end{array}$$

Next, it will be shown that the optimization problem (25) can be formulated as a pseudo-Boolean optimization (i.e., optimization of pseudo-Boolean functions). A pseudo-Boolean function is a mapping from a finite number of Boolean variables to a real number and can be uniquely represented by a multi-linear polynomial (Boros and Hammer, 2002).

As mentioned before, any logical matrix can be expressed by a multi-linear polynomial. After calculation, the term $\overset{T}{\underset{t=0}{\sum }}\overset{n}{\underset{i=1}{\sum }}x_{i}^{\text{T}}\left(t\right){\hat{x}}_{i}\left(\theta ,t\right)$ can be represented by a multivariate polynomial.

(26) $$\begin{array}{r} P_{mv}\left(\theta \right)=c+\underset{Q_{\beta }\subset V}{\sum }c_{Q_{\beta }}\underset{j\in Q_{\beta }}{\prod }\theta_{j}^{r_{Q_{\beta },j}} \end{array}$$

where $c,c_{Q_{\beta }}\in \mathbb{R}$ for $Q_{\beta }\subset V=\left\{1,2,\cdots \;,k\right\}$ and the factor $r_{Q_{\beta },j},\forall \beta ,j$ is a natural number. In addition, using the property of Boolean variables $\theta_{i}^{r}=\theta_{i},\forall r\in {\mathbb{Z}}_{+}$, the multivariate polynomial (26) is easily transformed into a multi-linear polynomial. Consequently, the term $\overset{T}{\underset{t=0}{\sum }}\overset{n}{\underset{i=1}{\sum }}x_{i}^{\text{T}}\left(t\right)\cdot {\hat{x}}_{i}\left(\theta ,t\right)$ can be described by a multi-linear polynomial (6) and the optimization problem (25) is transformed into a pseudo-Boolean optimization problem

(27) $$\begin{array}{r} \underset{\theta \in D^{k}}{\max}P_{ml}\left(\theta \right)=\underset{\theta \in D^{k}}{\max}c+\overset{k}{\underset{i=1}{\sum }}c_{i}\theta_{i}+\overset{q}{\underset{\alpha =1}{\sum }}c_{I_{\alpha }}\underset{j\in I_{\alpha }}{\prod }\theta_{j} \end{array}$$

So far, several different ways to handle the nonlinear pseudo-Boolean optimization problems (27) exist, such as reduction to an equivalent linear or quadratic binary programming problem, branch-and-bound method, linear approximations (Boros and Hammer, 2002; Crama and Rodrí-guez-Heck, 2017). As the linear programming relaxation of an integer linear program can be solved efficiently and based on the solution integer solutions can be found, in this paper we consider “linearization”, so that nonlinear binary optimization can be reduced to integer linear program (Crama and Rodrí-guez-Heck, 2017). The key is to introduce auxiliary Boolean variables $\text{z}={\left[z_{1}\;z_{2}\;\cdots \;\right]}^{\text{T}}$ to replace the nonlinear monomial $\prod_{j\in I_{\alpha }}\theta_{j}$ in (6) by means of the AND-expression $z_{\alpha }=\prod_{j\in I_{\alpha }}\theta_{j}$. Simultaneously to satisfy the AND-expression, linear inequalities as constraints are considered to get feasible value of the nonlinear monomial $\prod_{j\in I_{\alpha }}\theta_{j}$. Finally, an optimization problem equivalent to (27) is obtained as

(28) $$\begin{array}{l} \underset{\theta ,z}{\max}L_{P}(\theta ,z)=\underset{\theta ,z}{\max}c+\sum_{i=1}^{k}c_{i}\theta_{i}+\sum_{\alpha }c_{{ℐ}_{\alpha }}z_{\alpha }\\ \;s.t.\;z_{\alpha }\leq \theta_{j},\forall j\in {ℐ}_{\alpha },\\ \;z_{\alpha }\geq \sum_{j\in {ℐ}_{\alpha }}\theta_{j}-(|{ℐ}_{\alpha }|-1),\\ \;z_{\alpha }\in D,\;\theta \in D^{k}. \end{array}$$

The constraints in the optimization problem in (27) can be complemented by additional constraints representing the prior knowledge of alternative hypotheses or unateness as shown in Section 4.1 and Section 4.3, respectively.

Remark 2. *It is important to note that minimizing or maximizing a pseudo-Boolean function is known to be NP-hard (Crama and Rodrí-guez-Heck, 2017). However, Breindl et al. (2013) shows that the optimization problem (28) can be solved using a relaxed problem, i.e., linear programming solver based on the simplex method, which requires less computational effort than mixed integer linear program. The relaxed problem delivers an integer as optimal solution, which is also an optimal solution of the optimization problem (28)*.

### 5.2. Handling of large scale networks

With modern measurement techniques it is possible to quantify a huge amount of substances simultaneously. A Boolean network which describes the observed interactions is then also of large scale. But the number of substances which are direct relevant for the regulation of certain substance is usually limited, in other words the connectivity inside the network is bounded. For instance, as pointed out by Arnone and Davidson (1997), the connectivity is bounded by 8. Without prior knowledge the complexity of the algorithm is $O=2^{n+m}$ as all state and input combinations have to be considered as potential regulators for all states, even though only some of them are relevant in the end. This would limit the applicability of the approach to rather small networks. If one has hypotheses about potential interaction partners and the number of potential regulators per state is limited by a set of *k* variables, then the complexity of the algorithm is $O=2^{k}$, as the regulative functions for each state can be inferred separately. The hypotheses for the interaction partners are not necessarily based on prior-knowledge, but could also be computed based on the data set. In Margolin et al. (2006) an approach is presented, which is based on the information theoretic concept of mutual information ranking and the restriction to pairwise interactions that leads to a very good scaling with big data sets.

### 5.3. Handling of missing measurement values

Dependent on the measurement technique it is sometimes not possible to measure all states at all time instances and the missing values must be handled in the data analysis. There are approaches in the literature to compute an imputation e.g., for microarrays in Gan et al. (2006) and gel-based proteomics in Albrecht et al. (2010). These approaches are based on interpolation or heuristics. An alternative is to use a data analysis approach which can deal with incomplete data matrices.

A missing measurement value can be estimated during the identification by adding additional binary parameters in the identification process. Because of vector expression of states, all possible states belong to the set $\Delta_{2^{n}}$. In this way, *n* binary parameters are enough for vector expression of a completely unknown state at time *k*. For example, if *n* = 2, then we can generally express the unknown state as

(29) $$\begin{array}{r} x\left(k\right)=\left[\begin{array}{c} \gamma_{1}\cdot \gamma_{2}\\ \gamma_{1}\cdot \left(1-\gamma_{2}\right)\\ \left(1-\gamma_{1}\right)\cdot \gamma_{2}\\ \left(1-\gamma_{1}\right)\cdot \left(1-\gamma_{2}\right) \end{array}\right]. \end{array}$$

Furthermore, as the states of the system are known partially, then the number of binary parameters can be reduced accordingly. So for each missing value one parameter is added to the optimization and the imputation for this value is calculated which fits best to the other dynamic behavior of the system.

### 5.4. Handling of unmeasurable processes

In some systems post transcriptional protein-protein interactions induce dependencies between the measured abundances similar to the transcriptional regulation. This leads to the situation that the transcriptional regulation can not be observed directly and the identification procedure needs to be adapted accordingly (Geier et al., 2007). The dependencies between the states and the measured outputs can be included in boolean models easily by adding Boolean functions mapping from the actual stats *X*(*t*) to the measured outputs *Y*(*t*):

(30) $$\begin{array}{r} Y_{j}\left(t\right)=h_{j}\left(X\left(t\right)\right),\;j=1,2,\ldots ,p \end{array}$$

where ${\left[Y\left(t\right)=Y_{1}\left(t\right)Y_{2}\left(t\right)\;\ldots Y_{p}\left(t\right)\right]}^{T}\in D^{p}$ is the output vector at time *t*, *h*_*i*_ are logic functions. All structural information on the logic functions can be expressed with a logical matrix *H*

(31) $$\begin{array}{r} y\left(t\right)=Hx\left(t\right) \end{array}$$

which can be derived analogous to Equations (2–5). All approaches presented in this paper can be extended for the BN model with output mapping. As additional logic functions are to be identified, additional unknown parameters are added and these parameters cannot be separately identified from the parameters of the regulative functions, which impacts the computational burden drastically (Zhang et al., 2017b).

### 5.5. Influence of noise

In real world experiments measurement noise is unavoidable. With a sophisticated binarization method the influence of additive noise can often be suppressed (Hopfensitz et al., 2012). But noise can still lead to wrong binarized values in some cases and consequently errors in the input to the identification method cannot be totally avoided. As the presented approach is based on an optimization, the network which optimally fits to the observed data is found. Inconsistent transitions caused by noise in the data set can be handled directly and lead to an identification result with a non-zero prediction error. If, due to noise, the observed transitions would lead to an identification result which is contradictory to prior knowledge, the identification approach ignores these transitions directly.

Example 6. Consider the BCN for oxidative stress response pathways with the PI3-Kinase-Akt pathway given in Sridharan et al. (2012).

(32) $$\{\begin{array}{ll} X_{1}(t+1) & =U(t)\wedge \neg X_{6}(t)\\ X_{2}(t+1) & =\neg X_{1}(t)\\ X_{3}(t+1) & =\neg X_{1}(t)\wedge (X_{5}(t)\vee X_{3}(t)\\ X_{4}(t+1) & =X_{1}(t)\wedge \neg X_{6}(t)\\ X_{5}(t+1) & =X_{4}(t)\vee \neg X_{3}(t)\\ X_{6}(t+1) & =X_{5}(t)\wedge (\neg X_{6}(t)\vee \neg X_{2}(t)) \end{array}$$

In the model, *X*_1_ represents stress reactive intermediaries, *X*_2_ transcription factor A, *X*_3_ key protein, *X*_4_ protein kinase, *X*_5_ transcription factor B, *X*_6_ anti-stress response element, *U* stress signal. Using STP, (32) can be converted into the algebraic form (5) with $x\left(t\right)={⋉}_{i=1}^{6}x_{i}\left(t\right)\in \Delta_{64},u\left(t\right)\in \Delta_{2}$.

**Figure 2:**
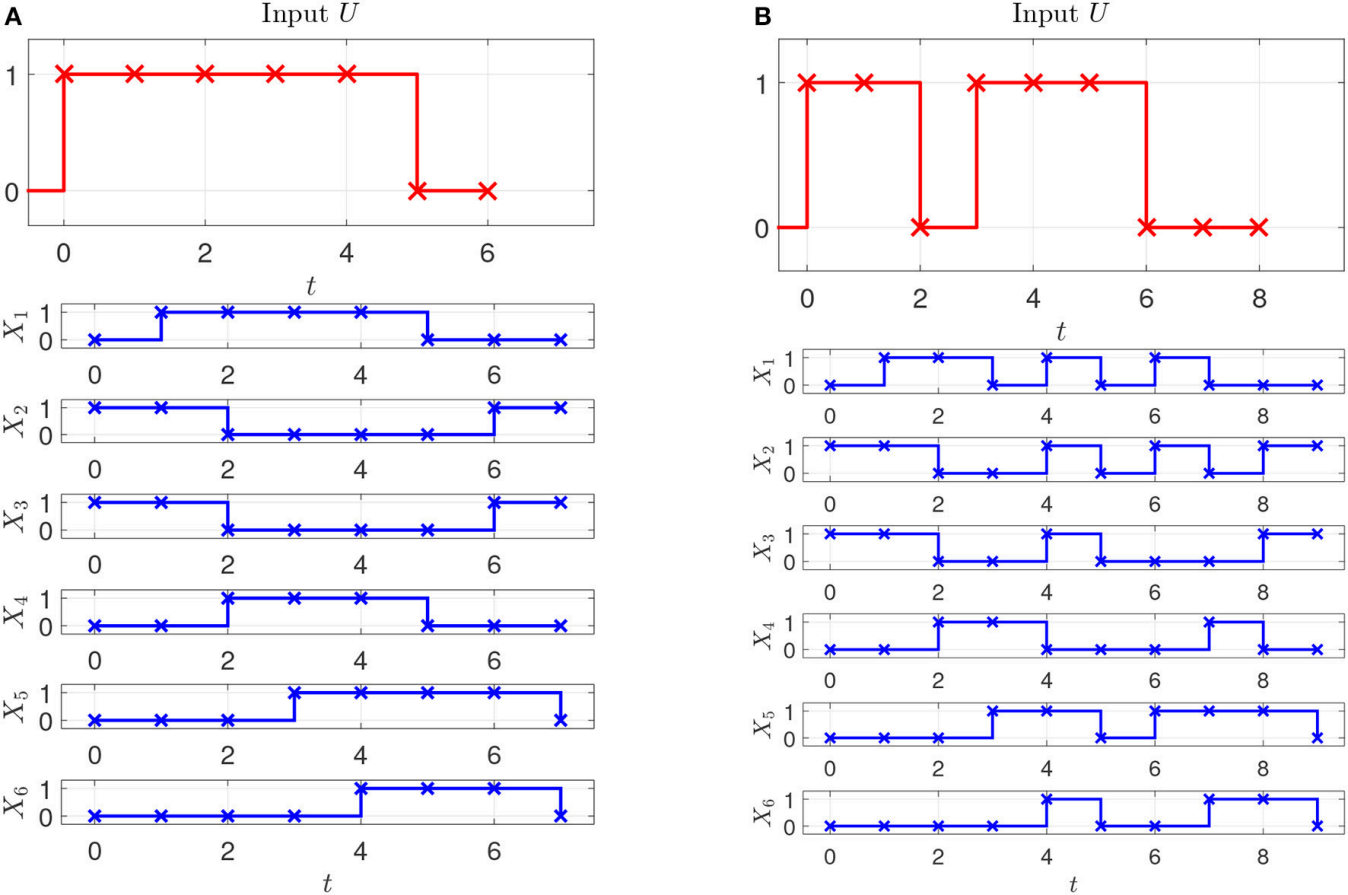
Perturbation and state measurement. **(A)** First experiment. **(B)** Second experiment.

**Figure 3:**
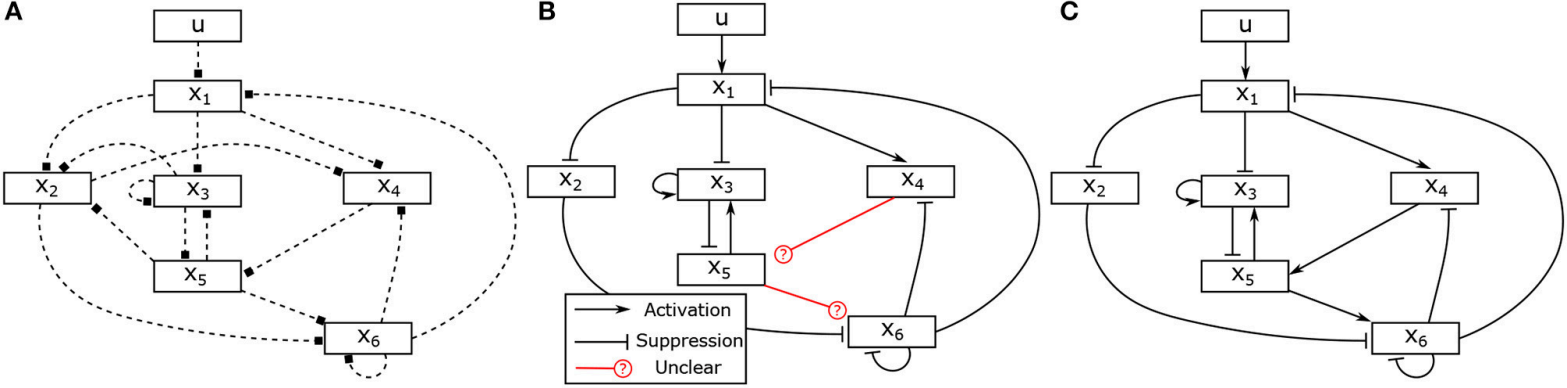
Hypothesis, partially identified and fully identified network graph. **(A)** Hypotheses for regulative interactions. **(B)** Identified Boolean network without canalizing information. **(C)** Identified Boolean network with prior knowlege.

Assume that two experiments have been executed starting in steady state with two different stimuli, the corresponding input-state data is obtained as shown in Figures 2A,B. Assume further that as prior knowledge the candidates of regulative interactions (see the dashed lines in Figure 3A) and the attractor are given. The attractor of the BCN without stress is *X*_1_ = 0, *X*_2_ = 1, *X*_3_ = 1, *X*_4_ = 0, *X*_5_ = 0, *X*_6_ = 0.

Based on the candidates of regulative interactions, the number of unknown binary parameters **θ** representing the logical matrices of the Boolean functions can be reduced from 6·2^7^ = 768 to 40 as described in Section 4.1. For instance, since the variable *X*_2_ is connected with the variables *X*_1_, *X*_3_ and *X*_5_, it means that the Boolean function of the variable *X*_2_ can be described by *f*_2_(*X*_1_, *X*_5_, *X*_5_). Accordingly, 8 binary parameters are enough to represent the logical matrix *S*_2_ of the Boolean function *f*_2_, i.e.,

(33) $$\begin{array}{r} S_{2}=\left[\begin{array}{cccccccc} \theta_{1} & \theta_{2} & \theta_{3} & \theta_{4} & \theta_{5} & \theta_{6} & \theta_{7} & \theta_{8}\\ 1-\theta_{1} & 1-\theta_{2} & 1-\theta_{3} & 1-\theta_{4} & 1-\theta_{5} & 1-\theta_{6} & 1-\theta_{7} & 1-\theta_{8} \end{array}\right]. \end{array}$$

The information about the steady state is used as described in Section 4.4 to determine one parameter in each matrix, which reduces the number of unknown variables to 34. In the next, we apply the proposed approach to identify the model of the BCN from the given input-state data. Solving the optimization problem (28), in total, 31 unknown binary parameters can be determined. The identification result is depicted in Figure 3B and the identified matrices are as follows,

(34) $$\begin{array}{l} S_{1}=\left[\begin{array}{cccc} 0 & 1 & 0 & 0\\ 1 & 0 & 1 & 1 \end{array}\right],\;S_{2}=\left[\begin{array}{cccccccc} 0 & 0 & 0 & 0 & 1 & 1 & 1 & 1\\ 1 & 1 & 1 & 1 & 0 & 0 & 0 & 0 \end{array}\right],\;\\ \;S_{3}=\left[\begin{array}{cccccccc} 0 & 0 & 0 & 0 & 1 & 1 & 1 & 0\\ 1 & 1 & 1 & 1 & 0 & 0 & 0 & 1 \end{array}\right],\\ S_{4}=\left[\begin{array}{cccccccc} 0 & 1 & 0 & 1 & 0 & 0 & 0 & 0\\ 1 & 0 & 1 & 0 & 1 & 1 & 1 & 1 \end{array}\right],\;S_{5}=\left[\begin{array}{cccc} \theta_{\text{29}} & 0 & 1 & 1\\ 1-\theta_{\text{29}} & 1 & 0 & 0 \end{array}\right],\;\\ \;S_{6}=\left[\begin{array}{cccccccc} 0 & 1 & \theta_{\text{35}} & 0 & 1 & 1 & \theta_{\text{39}} & 0\\ 1 & 0 & 1-\theta_{\text{35}} & 1 & 0 & 0 & 1-\theta_{\text{39}} & 1 \end{array}\right]. \end{array}$$

It can be seen that the logical matrices of the Boolean functions for *X*_5_ and *X*_6_ can not be uniquely determined. Combined with an additional information about activating or suppressing properties of the states, for instance, *X*_4_ and *X*_5_ are, respectively, activator to *X*_5_ and *X*_6_, the complete model can be uniquely reconstructed. The canalizing property of *X*_4_ and *X*_5_ can be utilized as described in Section 4.2. If this information is not available, one could conduct additional experiments with different stimuli and combine the data to have full reconstruction of the model as depicted in Figure 3C.

## 6. Discussion

The proposed method facilitates the incorporation of various types of prior knowledge. The optimization problem can be solved by efficient linear programming solvers. By using the simplex method one can guarantee to find the network which optimally fits to the observed data. In comparison, the genetic algorithms based approaches may not guarantee the optimal solution. The proposed method is developed for synchronous Boolean networks. It can be applied to large scale networks, if the connectivity of the network to be identified is limited with aid of prior knowledge or application of information theory.

In future we plan to investigate data-based approaches to infer the connections in large networks and automated partitioning into smaller subsystems (e.g., with an adapted approach from discrete event systems like Saives et al., 2018). We also work on a new method for the binarization based on the idea that the qualitative system behavior before and after the binarization shall be the same.

## Author contributions

TL, ZZ, and PZ conception and design of research. TL and ZZ performed simulation and analyzed data. TL, ZZ, and PZ interpreted simulation results. TL and ZZ prepared figures. TL, ZZ, and PZ drafted manuscript and approved final version of manuscript.

### Conflict of interest statement

The authors declare that the research was conducted in the absence of any commercial or financial relationships that could be construed as a potential conflict of interest.
